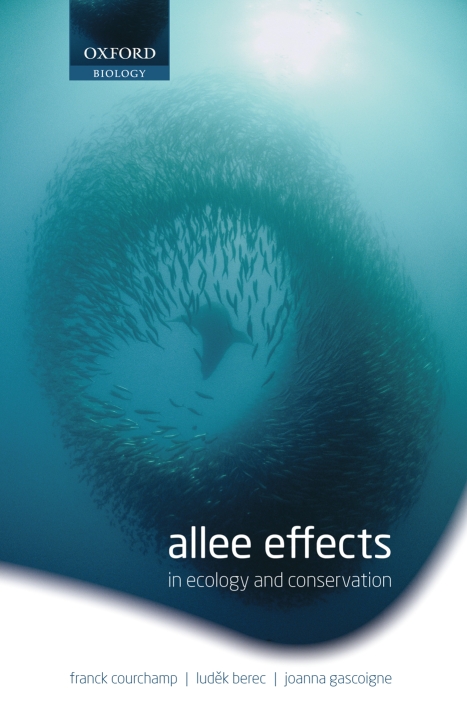# The Allure of the Few

**DOI:** 10.1371/journal.pbio.0060127

**Published:** 2008-05-20

**Authors:** Barry W Brook

## Abstract

Barry Brook reviews a new book on "Allee effects"--the processes by which small population sizes beget an accelerating decline in abundance and so hasten extinction.

Population ecology—the science of what makes animal and plant populations change, persist, or go extinct—is the most theoretical and mathematical of all ecological disciplines, yet it yields a huge number of practical benefits. When conservation agencies wish to protect a threatened species, but are up against a tight budget and real-world logistical constraints, they use population models to tell them how large an area to reserve and how it should be connected to other habitats, so as to minimise the species' risk of extinction. In short, population ecology tells us how to get the best “bang for our buck” [1]. Similarly, fishery managers face the perennial issue of determining how heavily they can exploit a fluctuating fish stock without over-harvesting and causing a crash (or extinction). Again, population models, of various complexities, provide guidance in setting fishing quotas and no-take areas [2].

The balance that must always be struck is model complexity. If population models are too simple, they risk overlooking key biological features and therefore giving biased predictions. Conversely, if they are too complex, it becomes difficult or impossible to reliably estimate the value of model components (parameters). For instance, the most fundamental “law” in population ecology states that changes in the size of populations are determined by the balance of birth rates, death rates, immigration, and emigration. Further abstractions can yield exponential growth models, doubling times and estimates of per-generational change, while additional complications might include demographic and environmental fluctuations, age structure, and a self-reinforcing population decline as a population approaches extinction [3]. Do these simplifications or complications matter when it comes to the practical business of threatened species conservation, pest control, or the setting of harvest quotas [2]?

One area where this question makes a big difference is in the “regulation” of populations. Why don't populations simply grow forever, and what stops an inevitable crash to extinction once they start to decline [4]? A simple assumption is that environmental fluctuations, driven by weather or predators, act to control population dynamics. A more complex model might reflect a limited supply of resources, such as food or living space, so that populations expand when few individuals are seeking access to shared resources and decline when competition among individuals for diminishing resources occurs at high densities. In the former case, a population can become extinct by chance, if its environment is highly variable or if it happens to suffer a few bad years in a row. In the latter case, a population is inherently more resilient, because populations tend to rebound from low densities. The relative importance of a random environment versus resource competition will strongly dictate whether a rare species persists, or the degree to which a game species or fishery can tolerate a harvest and still replace itself [4].

But what if neither of these assumptions is reasonable? It is easy enough to imagine situations in which low densities just make things progressively worse. This effect may be due to generic impacts like inbreeding or limitations on the availability of mates due to geographic dispersion, or species-specific traits such as the breakdown of anti-predator herd guarding or the failure of male bird breeding arenas (called leks) to attract females if they are too small. Such problems, where low numbers of individuals in a population leads to a self-perpetuating loss of demographic, genetic, or ecological fitness, are called “Allee effects”. This issue, and its rich ecological, evolutionary, and practical conservation implications, are explored in a new book by Franck Courchamp, Lud k Berec, and Joanna Gascoigne called *Allee Effects in Ecology and Conservation* [3].

The book spends a few chapters considering the historical and theoretical roots of the Allee effect (the study of which was instigated by behavioural biologist Warder C. Allee in the 1930s). It then leads naturally to a description, replete with examples, of the many behavioural, genetic, demographic, or ecological mechanisms, some described above, that have been shown to lead to “inverse density dependence” or “depensation” (as Allee effects are alternatively called in the entomological and fisheries literature, although these terms embody exactly the same concept). Some interesting real-world applications the authors describe in detail include: (1) the importance of incorporating Allee effects in population viability analysis (extinction-risk simulation models), and the insights gained by treating fisheries' exploitation of Norwegian herring as predation; (2) the way in which Allee effects can guide suppression of pest species (there is an optimum eradication threshold that most effectively minimises the likelihood of an outbreak for each dollar spent on control); (3) the reason for the regular failure of biological control measures (too few propagules of the control organism tend to be introduced); and (4) the stunning economic Allee effect whereby poachers or collectors place ever-greater value on attractive species as they are plundered and become rarer—the allure of the few [5].

The important mathematical architecture required to actually include Allee effects in population models is well-covered and clearly explained, but deliberately restricted to a single chapter. The stated intention was to make the other five chapters more accessible to a broad, non-technical readership. This works on one level, but it forced me to flip regularly back and forth through the book when reading the later chapters on genetics, evolution, conservation, and management as I tried to link the “hard-core theory” back to the concepts and examples (described above). The weakest section of the book was the description of the methods applied to detect Allee effects using empirical data—model fitting, observations of fitness, and time series of population size. The two case studies given here, on the Atlantic grand banks fisheries and invading gypsy moths in the north-eastern United States, failed to satisfactorily work through the technical stages of statistical fitting, interpretation, and model selection that must be used to decide on the strength of evidence for Allee effects. The authors do hint that this area is poorly developed in the literature, and I suspect they are right—this is a topic ripe for more research. But a clear, step-by-step illustration would have helped greatly.

I should add that the book is delightfully engaging to read. The authors write in a relaxed, almost mischievous style, which had the effect of making me feel like I was deliberating these topics with the authors over our beverage of choice rather than cogitating a dry tome in quiet isolation. This does not detract from the academic quality of the product—they present a strong mix of experimental, mensurative, and theoretically oriented mathematical examples. But their enthusiasm for the topic is infectious, which means the book is one of those rare ecology texts that can be enjoyably read, quickly, from cover to cover. It should also be of interest to non-ecologists, because a real effort is made to connect the broader concept of Allee effects to other sciences, including the study of human social behaviour. As the authors illustrate in their tongue-in-cheek foreword, a decision to attend a party might depend on whether your friends are going—but of course if everyone thinks this way, no one ends up attending for fear of being the only one! Other more serious examples include the breakdown and eventual collapse of rural societies after the relocation of a critical level of essential infrastructure in small towns to cities by centralised governments for administrative convenience, or the “extinction” of local languages as “invasive species” like English become more pervasive, causing native speakers to become rarer and making these languages less attractive or useful to learn.

But getting back to ecological implications of Allee effects, most population models used for wildlife management have, to date, tended to ignore them—probably because in most cases managers have had no information on how these effects may operate, or how important they are likely to be. Yet the clear take-home message from the comprehensive overview provided by *Allee Effects in Ecology and Conservation* [3] is that it is no longer tenable to dismiss Allee effects as an optional, “bolt-on” issue. Indeed, the authors of the book advocate quite the opposite—Allee effects are nearly ubiquitous, and almost any species one could envisage is likely to experience Allee effects at some time or place. The real challenge ahead is for enough studies to emerge to allow firm rules of thumb to be developed—generalisations on the circumstances under which Allee effects are most or least important in determining population viability and extinction risk. This is the sort of information and examples that conservation practitioners really need to know about, if they are to be convinced that Allee effects should be built into the models they use to manage real-world populations. With their new book, Courchamp, Berec, and Gascoigne provide a broad intellectual grounding and literature review upon which the future study and application of Allee effects, in all their varied guises, is sure to flourish.

**Figure:**